# Supplementary material for: Early Social Experience Affects the Development of Eye Gaze Processing
Source: Curr Biol. 2015 Dec 7;25(23):3086–91. doi: 10.1016/j.cub.2015.10.019 (PMC4683081; doi:10.1016/j.cub.2015.10.019)
Supplement: Document S1. Figure S1 [file mmc1.pdf]

Current Biology
Supplemental Information

Early Social Experience Affects the Development of Eye Gaze Processing

Atsushi Senju, Angélina Vernetti, Natasa Ganea, Kristelle Hudry, Leslie Tucker, Tony Charman, and Mark H. Johnson

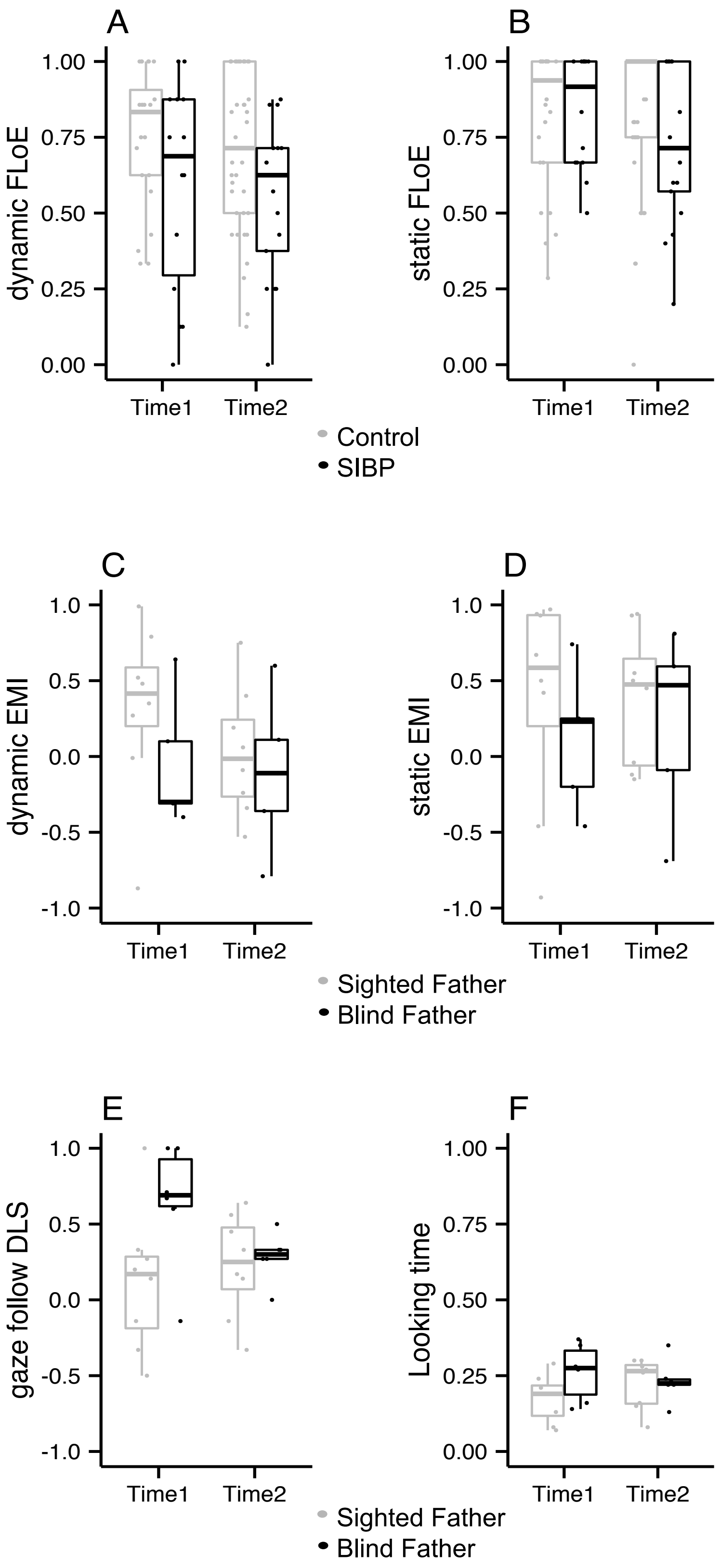

Figures S1, Related to Figure 1. A-B: Analyses of the first look. For dynamic (A) and static (B) condition of the face scanning task, proportions of first look on the eyes

(FLoE) were calculated by dividing the number of trials participants fixated on the eyes before the mouth by the total number of valid trials. As in the main analyses, each of 8 trials were excluded if less than one second of data was accumulated. In the dynamic condition, FLoE was significantly smaller in the SIBP group than in control group ($F(1, 35) = 4.18$, $p = .049$, $\eta_p^2 = .11$), suggesting that SIBP group were less likely to fixate on the eyes before the mouth compared to the control groups when they observe dynamic face. FLoE did not differ between groups in the static condition ($F(1, 35) = .44$, $p = .510$, $\eta_p^2 = .01$). Neither the main effect of visit nor the interaction between the group and the visit reached significance in either dynamic or static condition (all $F < 1.70$, all $p > .180$, all $\eta_p^2 < .06$).
C-F: Results of subgroup analyses of eye-tracking measurements in sighted infants of blind parents (SIBP), which contrast those children with blind fathers (n = 6) and those with sighted fathers (n = 8) in (C,D) the face scanning task, eye–mouth index (EMI) in (C) dynamic and in (D) static conditions, in (E, F) the gaze-following task, (E) differential looking score (DLS) and (F) looking time. Note that we did not find any significant main effect of group (all $F < 2.24$, all $p > .166$, all $\eta_p^2 < .18$) or group by visit interaction (all $F < 3.71$, all $p > .083$, all $\eta_p^2 < .27$) on the variables which showed significant difference between SIBP and controls. The main effect of group ($F(1, 8) = 19.03$, $p = .002$, $\eta_p^2 = .70$) and the interaction between group and visit ($F(1, 8) = 5.38$, $p = .049$, $\eta_p^2 = .40$) were significant in DLS, suggesting that SIBP with both blind mother and father show more frequent gaze following at time 1, but no further interpretations were made as we did not find any group differences between SIBP and controls on this variable.
The upper whisker extends from the hinge to the highest value that is within 1.5 * IQR of the hinge (where IQR is the inter-quartile range, or distance between the first and third quartiles). The lower whisker extends from the hinge to the lowest value within 1.5 * IQR of the hinge. Individual data points were also plotted on top of the box plots.
