# Supplementary material for: Early Social Experience Affects the Development of Eye Gaze Processing
Source: Curr Biol. 2015 Dec 7;25(23):3086–91. doi: 10.1016/j.cub.2015.10.019 (PMC4683081; doi:10.1016/j.cub.2015.10.019)
Supplement: Document S2. Article plus Supplemental Information [file mmc2.pdf]

Report

Current Biology

Early Social Experience Affects the Development of Eye Gaze Processing

Highlights

- Infants of blind parents allocate less attention to adults' eyes and gaze direction
- Differences in gaze processing in these infants become greater after 12 months of age
- Overall social communication skills development of them were within the typical range

Authors

Atsushi Senju, Angélina Vernetti, Natasa Ganea, Kristelle Hudry, Leslie Tucker, Tony Charman, Mark H. Johnson

Correspondence

a.senju@bbk.ac.uk

In Brief

Senju et al. show that infants of blind parents allocate less attention to adults' eyes and to the object adults are looking at, despite showing typical overall social communication. The results highlight that human functional brain development shows selective experience-dependent plasticity attuned to the individual's specific environment.

Senju et al., 2015, Current Biology *25*, 3086–3091
December 7, 2015 ©2015 The Authors
http://dx.doi.org/10.1016/j.cub.2015.10.019

Current Biology
Report

Early Social Experience Affects the Development of Eye Gaze Processing

Atsushi Senju,[1,*] Angélina Vernetti,[1] Natasa Ganea,[1] Kristelle Hudry,[2] Leslie Tucker,[1] Tony Charman,[3] and Mark H. Johnson[1]
[1]Centre for Brain and Cognitive Development, Birkbeck, University of London, Malet Street, London WC1E 7HX, UK
[2]Olga Tennison Autism Research Centre, School of Psychology and Public Health, La Trobe University, Melbourne, VIC 3086, Australia
[3]Department of Psychology, Institute of Psychiatry, Psychology & Neuroscience, King's College London, P.O. Box 077, De Crespigny Park, London SE5 8AF, UK
*Correspondence: a.senju@bbk.ac.uk
http://dx.doi.org/10.1016/j.cub.2015.10.019
This is an open access article under the CC BY license (http://creativecommons.org/licenses/by/4.0/).

SUMMARY

Eye gaze is a key channel of non-verbal communication in humans [1–3]. Eye contact with others is present from birth [4], and eye gaze processing is crucial for social learning and adult-infant communication [5–7]. However, little is known about the effect of selectively different experience of eye contact and gaze communication on early social and communicative development. To directly address this question, we assessed 14 sighted infants of blind parents (SIBPs) longitudinally at 6–10 and 12–16 months. Face scanning [8] and gaze following [7, 9] were assessed using eye tracking. In addition, naturalistic observations were made when the infants were interacting with their blind parent and with an unfamiliar sighted adult. Established measures of emergent autistic-like behaviors [10] and standardized tests of cognitive, motor, and linguistic development [11] were also collected. These data were then compared with those obtained from a group of infants of sighted parents. Despite showing typical social skills development overall, infants of blind parents allocated less attention to adult eye movements and gaze direction, an effect that increased between 6–10 and 12–16 months of age. The results suggest that infants adjust their use of adults' eye gaze depending on gaze communication experience from early in life. The results highlight that human functional brain development shows selective experience-dependent plasticity adaptive to the individual's specific social environment.

RESULTS

In two eye-tracking experiments, sighted infants of blind parents (SIBPs) and control infants watched simple actions on a computer screen while their gaze direction was continuously recorded. The face-scanning task [8] involved videos of female faces displaying dynamic actions, following a baseline period in which actresses remained still. An eye-mouth index (EMI) was calculated as (looking time to the eyes − looking time to the mouth)/(total looking time to the eyes and mouth). Higher EMI reflects a higher bias to look toward an adult's eyes. The gaze-following task [7, 9] presented a video of an actress shifting her gaze to one of two toys. We measured how frequently infants followed the adult's gaze and how long they looked at the gazed-at toy after following her gaze. Frequency of communication behavior was coded during naturalistic interaction between infants with their blind mothers and separately with a sighted unfamiliar adult (i.e., experimenter). The Autism Observation Scale for Infants (AOSI) [10] was used to assess emergent autistic-like behavior, and the Mullen Scales of Early Learning (MSEL) [11] were used as a standardized assessment of general developmental ability.

A series of group (SIBPs versus controls) by visit (time 1 versus time 2) repeated-measures ANOVAs revealed that SIBPs looked at adults' faces differently from controls. SIBPs had lower EMI when they observed dynamic faces (Figure 1A; main effect of group, $F(1, 31) = 5.92$, $p = 0.021$, $\eta_p^2 = 0.16$), demonstrating that they looked less at adults' eyes relative to mouth. A similar trend was observed in the EMIs when viewing static faces, though this did not reach significance (Figure 1B; main effect of group, $F(1, 32) = 4.02$, $p = 0.054$, $\eta_p^2 = 0.11$). Similarly, SIBPs were less likely to fixate on the eyes before the mouth in the dynamic condition, but not in the static condition (Figures S1A and S1B). In the gaze-following task, SIBPs followed the actor's gaze as frequently as control infants (Figure 1C; main effect of group, $F(1, 35) = 1.09$, $p = 0.305$, $\eta_p^2 = 0.03$), but they showed shorter looking time on the gaze-cued object than control infants (Figure 1D; main effect of group, $F(1, 34) = 7.08$, $p = 0.012$, $\eta_p^2 = 0.17$). These results show that SIBPs are less likely to attend to an adult's eye movement and less likely to then use the adult's gaze direction to control their own allocation of attention. For all of four eye-tracking measurements, neither the main effect of visit nor group by visit reached significance (all $F < 2.79$, all $p > 0.103$, all $\eta_p^2 < 0.08$). There was no evidence that these group differences were modulated by the amount of experience with sighted adults, such as having sighted versus blind father (see Figures S1C–S1F).

We also tested the hypothesis that these effects increase over the course of time as an infant's cortical system matures and gains more individualized experience [12, 13]. Results suggested

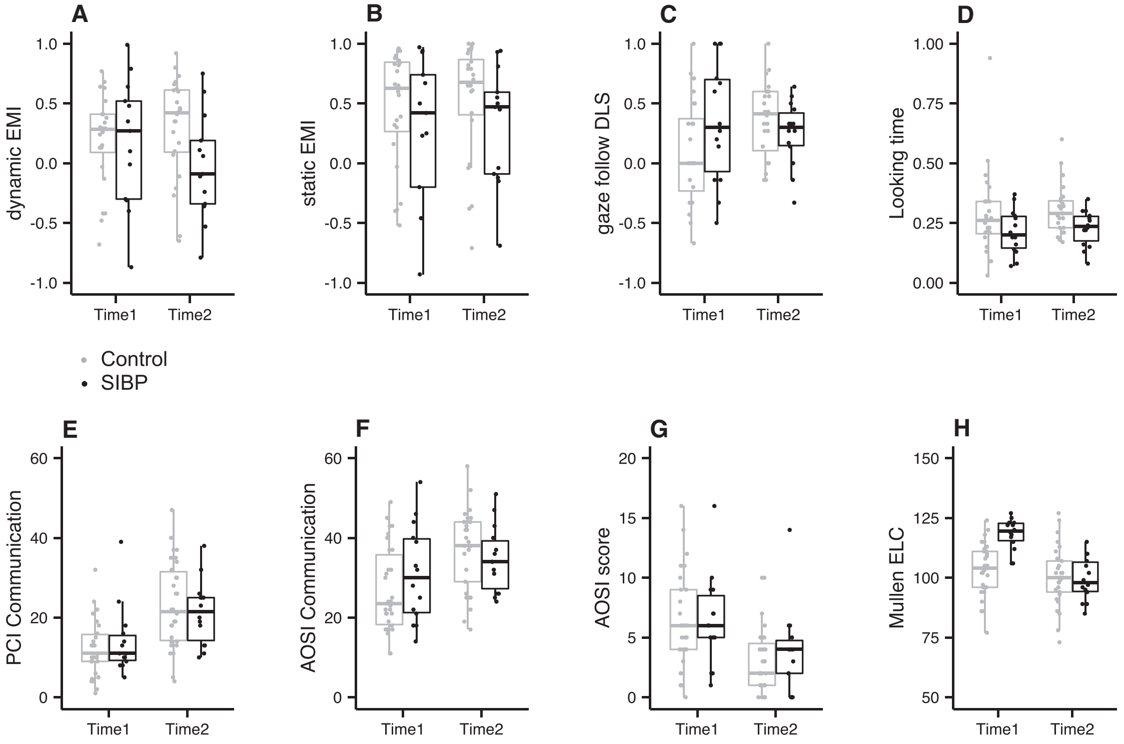

Figure 1. Results of SIBP and Control Infants

Results of sighted infants of blind parents (SIBP; black) and control infants (control; gray) at time 1 (6–10 months) and time 2 (12–16 months).

(A and B) The face-scanning task; eye-mouth index (EMI) in dynamic (A) and static (B) conditions.

(C and D) The gaze-following task; differential looking score (DLS) (C) and looking time (D).

(E and F) Frequency of communication events; the parent–child interaction (PCI) (E) and the Autism Observation Scale for Infants (AOSI) with a sighted examiner (F).

(G) AOSI total score.

(H) Mullen early learning composite (ELC) score.

See also Figure S1. The upper whisker extends from the hinge to the highest value that is within 1.5 × the interquartile range (IQR) of the hinge, where IQR is the distance between the first and third quartiles. The lower whisker extends from the hinge to the lowest value within 1.5 × IQR of the hinge. Individual data were also plotted on top of boxplots as dots.

that the magnitude of group differences was larger at time 2 (12–16 months old) than at time 1 (6–10 months old). At time 2, SIBPs showed significantly lower EMIs for dynamic ($t(31) = 2.63$, $p = 0.013$, $d = 0.95$) and static ($t(17.4) = 2.41$, $p = 0.027$, $d = 1.16$) face stimuli, as well as significantly shorter looking time at the gaze-cued object in the gaze-following task ($t(34) = 2.61$, $p = 0.013$, $d = 0.89$). By contrast, none of these group differences in eye-tracking measurements reached significance (all $t < 1.41$, all $p > 0.167$, all $d < 0.49$) at time 1. Further, as in the main analyses, the frequency of gaze following did not differ between groups at either visit ($t < 1.61$, $p > 0.118$, $d < 0.55$). However, these comparisons should be treated with caution, as neither the group by visit interaction nor the main effect of visit reached significance (all $F < 2.79$, all $p > 0.104$, all $\eta_p^2 < 0.07$).

SIBPs did not show overall delay in social skills development at these age ranges and had similar frequency of communication behavior both with their blind parents (Figure 1E; main effect of group, $F(1, 40) = 0.04$, $p = 0.844$, $\eta_p^2 < 0.01$) and with an unfamiliar sighted adult (Figure 1F; main effect of group, $F(1, 40) = 0.11$, $p = 0.743$, $\eta_p^2 < 0.01$) as did controls, and they did not show elevated levels of emergent autistic-like behavior, including atypical social behavior, as coded by the AOSI (Figure 1G; no main effect of group, $F(1, 39) = 0.04$, $p = 0.838$, $\eta_p^2 < 0.01$). These infants manifested emergent social skills between visits 1 and 2, showing more communicative behavior and less autistic-like behavior at time 2 than at time 1 (main effect of visit, all $F > 11.71$, all $p < 0.01$, all $\eta_p^2 > 0.23$). These main effects were not modulated by group by visit interactions (all $F < 3.03$, all $p > 0.090$, all $\eta_p^2 < 0.07$).

SIBPs also showed different general developmental profiles, as measured by MSEL, compared to controls, evidenced in a significant group by visit interaction ($F(1, 40) = 11.81$, $p = 0.001$, $\eta_p^2 = 0.23$). At time 1, SIBPs showed significantly higher MSEL early learning composite (ELC) standard scores than control infants (Figure 1H; $t(40) = 4.51$, $p < 0.001$, $d = 1.43$), demonstrating more advanced developmental level. Follow-up analyses showed that this was mainly driven by higher Visual Reception ($t(36) = 4.98$, $p < 0.001$, $d = 1.66$) and Receptive Language ($t(34) = 5.82$, $p < 0.001$, $d = 1.99$) subscale scores. At time 2, by contrast, no group difference was observed in the ELCs (Figure 1H; $t(40) = 0.57$, $p = 0.570$, $d = 0.18$). As past studies indicated the possible relationship between language development and face scanning [8, 14, 15] or gaze

following [9, 16], we examined whether the individual differences in receptive language score predicted the eye-tracking measures but found no such relationship between these at either visit or in either group (all $R^2 < 0.15$, all $F < 3.37$, all $p > 0.080$).

DISCUSSION

While a recent case series report with SIBPs concluded that the individuals described did not show significant differences in eye gaze processing with a sighted observer in either video or live interactions [17], in the present study, containing the largest sample size and density of measures reported, we demonstrate that selectively different experience in eye contact and gaze communication with the primary caregiver specifically affects the development of eye gaze processing. Infants of blind parents looked less at an adult's eyes relative to their mouth and, further, allocated less attention to an object to which the adult was looking. The study also revealed that these effects are selective because infants of blind parents did not show differences in overall social skills development and communication behavior as assessed by the AOSI and through the coding of communication behaviors during interactions with blind parents and with sighted experimenters. SIBPs also showed typical eye gaze processing within simpler orienting tasks, such as orienting to an adult's gaze direction. These results suggest that the developmental atypicality among infants of blind parents is restricted to attentional engagement to dynamic eye movement and to objects that are cued by direction of eye gaze. Follow-up analyses also highlighted that these group differences were small and non-significant at 6–10 months of age (Cohen's d = 0.37–0.48) and only became large and significant at 12–16 months of age (d = 0.89–1.16), indicating that atypical experience of gaze communication does not have a major impact on initial eye gaze processing during the first year of life but rather has increasing developmental impact beyond the first birthday. However, these developmental changes need to be interpreted with caution, as neither the interaction between group and visit nor the main effect of visit reached statistical significance in our analyses.

It is important to stress that, unlike the current study, previously reported case series studies [8, 18–20], including that from our own group [17], have largely failed to identify atypicalities in social cognitive development among SIBPs. This may be partly because the key findings in the current study can only be readily detected with a conventional group comparison study and not by a less sensitive clinically oriented case series with a small sample size.

The implications of the current findings go well beyond characterizing the development of SIBPs and highlight the critical role of selective postnatal experience on functional brain development. Infants of blind parents diverged from the control group in their eye gaze processing, a result that is inconsistent with strong nativist accounts hypothesizing that the development of social information processing is to a large extent independent of postnatal experience [21]. The divergence was not generalized to overall social skills development or frequency of communication with their parents or an unfamiliar adult but is specific to attention to adults' eyes and gaze cueing. Our findings are consistent with the affective learning viewpoint [22–24], which hypothesizes that the acquisition of eye contact and gaze communication emerges as a result of extensive exposure to the co-occurrence of eye contact and a wide variety of positive experiences through social interaction and communication [25], or the effect of social reinforcement on the development of infants' gaze-following behavior [23, 24]. However, the emerging pattern of increasing divergence suggested by the planned comparisons at each age indicates that such an affective learning process may modulate the later-emerging specialization of such skills rather than the initial acquisition of eye gaze processing skills.

The present results are also consistent with the view that infants are born with initial predispositions to process their species-typical environment, which then also guide the later experience-dependent development of specialized cognition adaptive to the given individual environment [12, 13]. The differences in eye gaze processing we observed were of small effect size at 6–10 months of age, consistent with the claim that infants are predisposed to develop the tendency to orient toward human eyes and to follow gaze even with limited experience of gaze communication due to blindness of the primary caregiver. The large and significant group difference at 12–16 months of age is generally consistent with the later emergence of specialized development adaptive to the individual environment. However, note that we did not find significant interaction between group and visit. Data from even younger infants of blind parents are required to examine whether the initial acquisition of eye gaze processing skills is indeed typical.

The profile of overall general cognitive development we observed (as assessed by the MSEL) partly replicated our previous case series study [17]. Infants of blind parents showed significantly higher ELC scores than controls at time 1, but no group differences were found at time 2. A number of possible factors could have contributed to the group difference. At 6–10 months of age, the higher ELC scores are mainly driven by the Visual Reception subscale scores as well as the Receptive Language subscale. The test items used to assess visual reception at this age range include those related to visual attention and memory. Thus, it may be that the need to switch between visual, auditory, and tactile modes of communication enhances the development of executive attention, similar to that observed in bilingually exposed infants [26]. We further suggest that enhanced exposure to, and dependence on, auditory communication may have facilitated receptive language skills growth in this early period. Further studies will be required to investigate the general cognitive and motor skills development of infants of blind parents, including via experimental measurements that are better attuned to assess specific elements of cognitive and motor functioning.

There are several questions that merit future investigation. First, it is possible that the emerging divergence found in the SIBP group could result not only from reduced experience of eye gaze communication but also from an alternative mode of communication with blind parents that relies less on eye gaze or other visual cues. In addition, further research will need to include infants of parents with alternative sensory impairments and/or adopted modes of communication, such as parents who are deaf or who have motor impairments that impact upon communication. Second, and relatedly, SIBPs might be utilizing cues afforded via more gross communicative gestures, such as

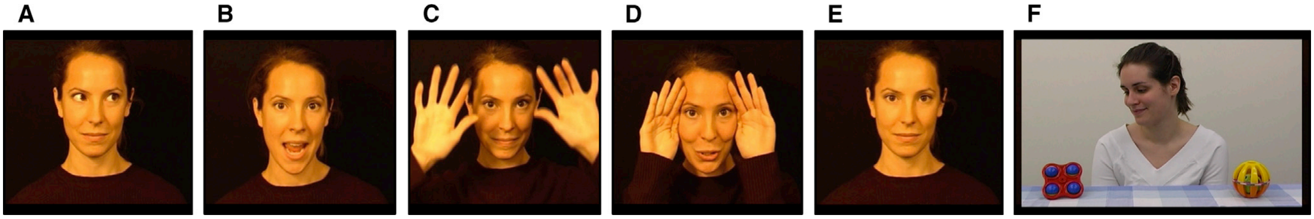

Figure 2. Selected Frames from the Stimuli

(A–E) Illustrations of actor's movements in eye movement (A), mouth movement (B), hand movement (C), peek-a-boo (coordinated movement of eyes, mouth, and hands) (D), and still face (E) in face-scanning task.

(F) Head turn to an object in gaze-following task.

head turns, which might explain their typical development of the frequency of following gaze (measured by differential looking score [DLS]). Further studies will benefit from studying the understanding of wider range of communicative gestures in this population. Finally, it will be important to understand the longer-term developmental implications of exposure to different early social experiences for SIBPs. Will SIBPs show even more divergent pattern of eye gaze processing over the course of life, or will this become more typical when they start to engage more regularly with sighted others—adults and peers—and spend less time with their blind primary caregiver? We are currently following up the current sample to find answers to this question in the future.

To conclude, the current results show that being reared with reduced experience of eye contact and gaze behavior from the primary caregiver has a selective effect on the later development of eye gaze processing. The development of SIBPs deviates from the norm in specific aspects of the processing of dynamic eye gaze, despite an overall typical pattern of development of social skills and social communicative behavior, and this becomes more prominent at 12–16 months of age. Our results highlight the critical role of experience-dependent learning that optimizes the brain to individually different salient aspects of the social environment.

EXPERIMENTAL PROCEDURES

Data from 14 sighted infants (seven males) of blind mothers were included in the final analyses. SIBPs were recruited through charities, online communities of parents, and personal contacts. All the blind mothers were the infants' primary caregivers. While the degree and the cause of visual impairment in the blind mothers varied, all had experienced profound visual impairment for at least 15 years at the time of infant testing, and the extent of their visual impairment severely affected face-to-face communication with their infants. Parent-infant dyads visited our center twice, once between 6 and 10 months (time 1, mean = 8.85, SD = 1.10) and then again between 12 and 16 months (time 2, mean = 14.28, SD = 0.88). Four additional dyads, who only completed a single visit, were not included in the analyses. A subset of the results from some of these infants (n = 5) were reported in a previous paper [17]. The assessment age points were predetermined to coincide with the availability of age-matched control data (n = 32, 14 males; time 1: mean age = 8.26, SD = 0.90; time 2: mean = 14.69, SD = 1.00) from infants of sighted parents [8, 9, 27], who also took part in the British Autism Study of Infant Siblings (BASIS, http://www.basisnetwork.org/) at the Centre for Brain and Cognitive Development, Birkbeck and completed two visits. The procedure was approved by the Research Ethics Committee of the School of Psychology, Birkbeck, University of London.

At each visit, infants completed two eye-tracking experiments of gaze processing and behavioral assessments of social communicative and cognitive development, and the dyads were recorded during naturalistic parent-child interaction (PCI). Data from these tasks were then compared with those from the same assessments conducted with the large group of sighted infants of sighted parents.

In the two experimental tasks, infants' looking behavior was recorded using an eye tracker (Tobii 1750 or T120, Tobii Technology). In the two standardized assessments and the PCI, recording was via digital video camera.

In the face-scanning task [8], infants were presented with videos of female faces displaying four different dynamic sequences, each lasting approximately 16 s: (1) the eyes displayed gaze shifts (Figure 2A), (2) the mouth displayed vowel articulation movements (Figure 2B), (3) the hands positioned near the face displayed an upward to downward motion (Figure 2C), and (4) the eyes, mouth, and hands moved together displaying a "peek-a-boo" sequence (Figure 2D). Each of these was preceded by a 5-s baseline period where the face was still (Figure 2E). Pseudorandom presentation continued for a maximum of eight total trials per infant (two per sequence). Areas of interest (AOIs) were defined around the eye and mouth region. Each of eight trials was excluded if less than one second of data was accumulated. An EMI was calculated as (looking time to the eyes − looking time to the mouth)/(total looking time to the eyes and mouth). EMIs were then averaged for the static baseline period and for the dynamic period.

In the gaze-following task [7, 9], infants observed a female actor seated in front of a table with two objects on top of it, one to the left and one to the right. The actor then turned her head to look at one of the objects (Figure 2F), with the direction of gaze counterbalanced across trials. Each infant viewed 12 trials. The DLS, which is commonly used to assess gaze-following behavior [5–7], was then calculated as the difference between the number of trials in which infants first looked at the object to which the actor gazed (i.e., the congruent object) and the trials in which infants looked at the other (i.e., incongruent) object. The number of incongruent trials was subtracted from the number of congruent trials, which was then divided by the sum of two types of trial to derive the DLS. To measure infants' attention to the congruent object, we averaged looking time on this object during those trials when the infant successfully followed the actor's gaze (i.e., looked first toward the congruent object).

Short periods of naturalistic PCI were video recorded in the lab. Dyads were given a box containing a small number of age-appropriate toys, and parents were asked to play as they normally would at home, using the toys if desired. Infant communication behaviors were later coded across a 6-min sample of the interaction, beginning when the researchers left the area and hid behind a curtain and the parent and infant were left alone to play. Each infant communication act was identified and coded based on the social communication protocol of Clifford et al. [28], resulting in a count of the total number of communication events directed toward the parent (including initiations and responses and whether signaled verbally or non-verbally). Coding of all footage was undertaken by two independent raters, blind to all information about participants (including group membership, age at visit, and all other data collected) and to the study aims and hypotheses. Inter-rater reliability was assessed for a random sample of control infants (17 clips) as well as the SIBP footage (10 clips), which was excellent (intra-class correlation coefficient [ICC] = 0.91).

The AOSI [10] is a semi-structured play assessment with an unfamiliar adult, originally designed to assess early behavioral atypicality in infants at familial high risk of autism. This was administered given reports of the increased

prevalence of autistic-like behaviors in blind children [29] and in children who have experienced severe environmental adversity in their early development [30]. We video recorded the AOSI assessments and analyzed 6-min samples within the free play periods to code the communication behavior between infants and the sighted adult examiner using the same protocol for coding PCI. As in PCI, the video footage was coded by a blinded independent rater, and subset of clips (3 clips of SIBPs and 13 clips of controls) were again double coded to check inter-rater reliability, which was very high (ICC = 0.82).

The MSEL [11] is a standardized, direct assessment of verbal and non-verbal abilities for children aged from birth to 6 years. It was used to assess the general developmental level of infants at each visit. Scores across four domains—Visual Reception, Fine Motor, Receptive Language, and Expressive Language—are combined to yield an overall ELC (M = 100, SD = 15).

All data were analyzed with a series of repeated-measures mixed-factorial ANOVAs with group (SIBPs versus controls) as a between-participants factor and visit (time 1 at 6–10 months old versus time 2 at 12–16 months old) as a within-participant factor. Individual data points that were either below (the first quartile − 1.5 × inter-quartile range [IQR]) or above (the third quartile + 1.5 × IQR) were removed from analyses as outliers, following Tukey [31]. When the assumption of homogeneity of variance was not supported, we corrected the statistics accordingly. We also removed individuals with missing data points from each ANOVA.

SUPPLEMENTAL INFORMATION

Supplemental Information includes one figure and can be found with this article online at http://dx.doi.org/10.1016/j.cub.2015.10.019.

AUTHOR CONTRIBUTIONS

Conceptualization, A.S. and M.H.J.; Methodology, A.S., K.H., T.C., and M.H.J.; Formal Analysis, A.S. and A.V.; Investigation, A.V., N.G., and L.T.; Data Curation, A.V., N.G., and K.H.; Writing – Original Draft, A.S.; Writing – Review & Editing, A.V., N.G., K.H., L.T., T.C., and M.H.J.; Visualization, A.V.; Project Administration, N.G. and L.T.; Funding Acquisition, A.S., T.C., and M.H.J.

ACKNOWLEDGMENTS

This work was supported by a UK Medical Research Council Career Development Award (G1100252) and a UK Economic and Social Research Council Research Fellowship (RES-063-27-0207) to A.S., the BASIS funding consortium led by Autistica (http://www.basisnetwork.org), and a UK Medical Research Council Programme Grant (G0701484 and MR/K021389/1) to M.H.J. The work was affiliated to the BASIS network, which provided the testing protocol and the access to the control data. We thank all the participating families, the Royal National Institute of Blind People (especially Sue Keil and Lauren Kelly), Mark Pimm, and Naomi Dale for kind support and Kim Davies, Issy Gammer, Holly Garwood, Sarah Hearne, Rebecca Kam, Helen Maris, Helena Ribeiro, and Erica Salomone for the help with testing and data coding.

Received: July 8, 2015
Revised: August 27, 2015
Accepted: October 6, 2015
Published: November 19, 2015

REFERENCES

1. Kleinke, C.L. (1986). Gaze and eye contact: a research review. Psychol. Bull. *100*, 78–100.
2. Pfeiffer, U.J., Vogeley, K., and Schilbach, L. (2013). From gaze cueing to dual eye-tracking: novel approaches to investigate the neural correlates of gaze in social interaction. Neurosci. Biobehav. Rev. *37*, 2516–2528.
3. Senju, A., and Johnson, M.H. (2009). The eye contact effect: mechanisms and development. Trends Cogn. Sci. *13*, 127–134.
4. Farroni, T., Csibra, G., Simion, F., and Johnson, M.H. (2002). Eye contact detection in humans from birth. Proc. Natl. Acad. Sci. USA *99*, 9602–9605.
5. Corkum, V., and Moore, C. (1998). The origins of joint visual attention in infants. Dev. Psychol. *34*, 28–38.
6. Moore, C., and Corkum, V. (1998). Infant gaze following based on eye direction. Br. J. Dev. Psychol. *16*, 495–503.
7. Senju, A., and Csibra, G. (2008). Gaze following in human infants depends on communicative signals. Curr. Biol. *18*, 668–671.
8. Elsabbagh, M., Bedford, R., Senju, A., Charman, T., Pickles, A., and Johnson, M.H.; BASIS Team (2014). What you see is what you get: contextual modulation of face scanning in typical and atypical development. Soc. Cogn. Affect. Neurosci. *9*, 538–543.
9. Bedford, R., Elsabbagh, M., Gliga, T., Pickles, A., Senju, A., Charman, T., and Johnson, M.H.; BASIS team (2012). Precursors to social and communication difficulties in infants at-risk for autism: gaze following and attentional engagement. J. Autism Dev. Disord. *42*, 2208–2218.
10. Bryson, S.E., Zwaigenbaum, L., McDermott, C., Rombough, V., and Brian, J. (2008). The Autism Observation Scale for Infants: scale development and reliability data. J. Autism Dev. Disord. *38*, 731–738.
11. Mullen, E.M. (1995). Mullen Scales of Early Learning - AGS Edition (AGS Publishing).
12. Johnson, M.H., and Munakata, Y. (2005). Processes of change in brain and cognitive development. Trends Cogn. Sci. *9*, 152–158.
13. Johnson, M.H., Senju, A., and Tomalski, P. (2015). The two-process theory of face processing: modifications based on two decades of data from infants and adults. Neurosci. Biobehav. Rev. *50*, 169–179.
14. Lewkowicz, D.J., and Hansen-Tift, A.M. (2012). Infants deploy selective attention to the mouth of a talking face when learning speech. Proc. Natl. Acad. Sci. USA *109*, 1431–1436.
15. Young, G.S., Merin, N., Rogers, S.J., and Ozonoff, S. (2009). Gaze behavior and affect at 6 months: predicting clinical outcomes and language development in typically developing infants and infants at risk for autism. Dev. Sci. *12*, 798–814.
16. Brooks, R., and Meltzoff, A.N. (2005). The development of gaze following and its relation to language. Dev. Sci. *8*, 535–543.
17. Senju, A., Tucker, L., Pasco, G., Hudry, K., Elsabbagh, M., Charman, T., and Johnson, M.H. (2013). The importance of the eyes: communication skills in infants of blind parents. Proc. Biol. Sci. *280*, 20130436.
18. Adamson, L., Als, H., Tronick, E., and Brazelton, T.B. (1977). The development of social reciprocity between a sighted infant and her blind parents. A case study. J. Am. Acad. Child Psychiatry *16*, 194–207.
19. Collis, G.M., and Bryant, C.A. (1981). Interactions between blind parents and their young children. Child Care Health Dev. *7*, 41–50.
20. Whaley, A. (1985). Visually impaired parents, sighted children - a bibliography. J. Vis. Impair. Blind. *79*, 196–197.
21. Zhu, Q., Song, Y., Hu, S., Li, X., Tian, M., Zhen, Z., Dong, Q., Kanwisher, N., and Liu, J. (2010). Heritability of the specific cognitive ability of face perception. Curr. Biol. *20*, 137–142.
22. Hood, B.M., Macrae, C.N., Cole-Davies, V., and Dias, M. (2003). Eye remember you: the effects of gaze direction on face recognition in children and adults. Dev. Sci. *6*, 67–71.
23. Corkum, V., and Moore, C. (1995). Development of joint visual attention in infants. In Joint Attention: Its Origins and Role in Development, C. Moore, and P.J. Dunham, eds. (Lawrence Erlbaum Associates, Inc.), pp. 61–83.
24. Triesch, J., Teuscher, C., Deák, G.O., and Carlson, E. (2006). Gaze following: why (not) learn it? Dev. Sci. *9*, 125–147.
25. Zeifman, D., Delaney, S., and Blass, E.M. (1996). Sweet taste, looking, and calm in 2- and 4-week-old infants: the eyes have it. Dev. Psychol. *32*, 1090–1099.
26. Kovács, Á.M., and Mehler, J. (2009). Cognitive gains in 7-month-old bilingual infants. Proc. Natl. Acad. Sci. USA *106*, 6556–6560.

27. Elsabbagh, M., Mercure, E., Hudry, K., Chandler, S., Pasco, G., Charman, T., Pickles, A., Baron-Cohen, S., Bolton, P., and Johnson, M.H.; BASIS Team (2012). Infant neural sensitivity to dynamic eye gaze is associated with later emerging autism. Curr. Biol. *22*, 338–342.

28. Clifford, S., Hudry, K., Brown, L., Pasco, G., and Charman, T.; PACT Consortium (2010). The Modified-Classroom Observation Schedule to Measure Intentional Communication (M-COSMIC): evaluation of reliability and validity. Res. Autism Spectr. Disord. *4*, 509–525.

29. Brown, R., Hobson, R.P., Lee, A., and Stevenson, J. (1997). Are there "autistic-like" features in congenitally blind children? J. Child Psychol. Psychiatry *38*, 693–703.

30. Rutter, M.L., Kreppner, J.M., and O'Connor, T.G.; English and Romanian Adoptees (ERA) study team (2001). Specificity and heterogeneity in children's responses to profound institutional privation. Br. J. Psychiatry *179*, 97–103.

31. Mcgill, R., Tukey, J.W., and Larsen, W.A. (1978). Variations of box plots. Am. Stat. *32*, 12–16.

Current Biology

Supplemental Information

Early Social Experience Affects the Development of Eye Gaze Processing

Atsushi Senju, Angélina Vernetti, Natasa Ganea, Kristelle Hudry, Leslie Tucker, Tony Charman, and Mark H. Johnson

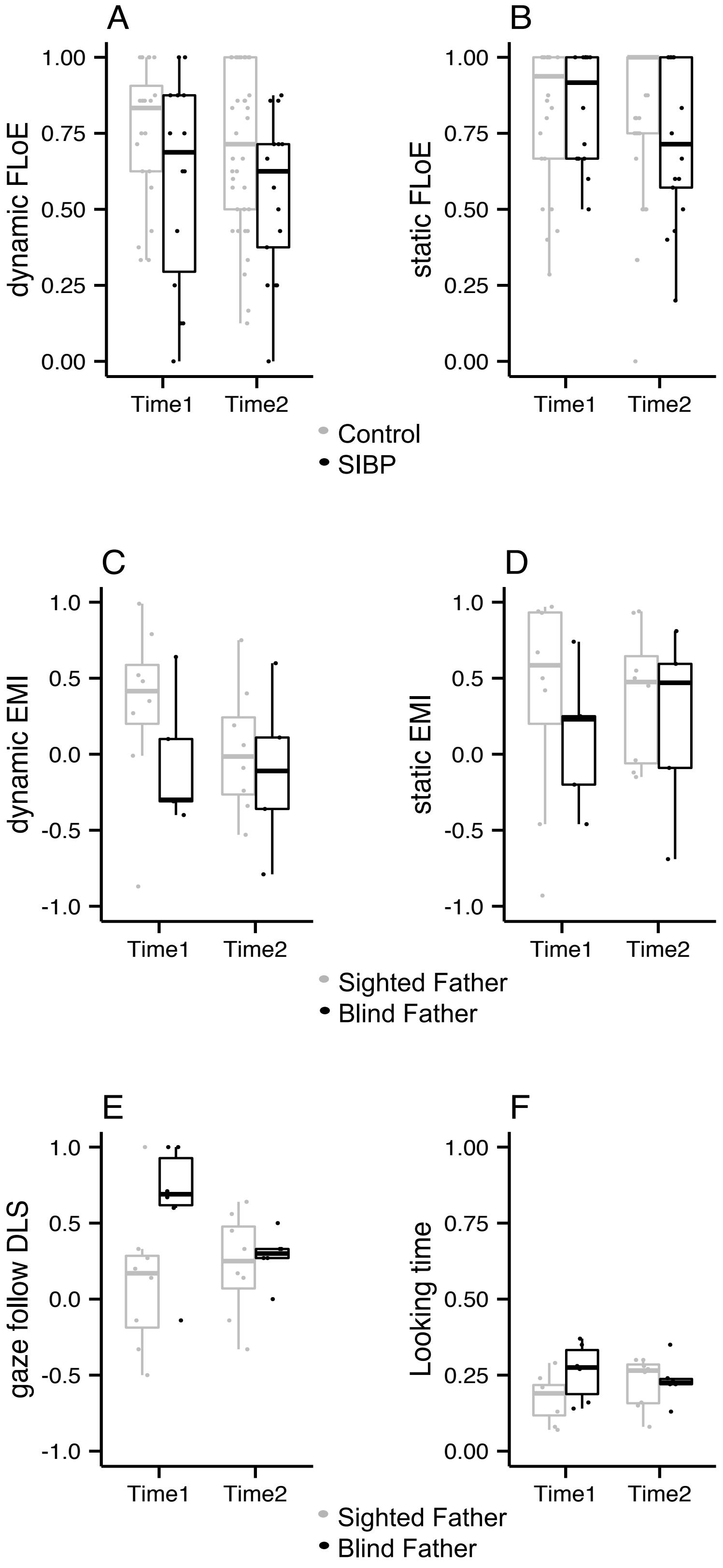

Figures S1, Related to Figure 1. A-B: Analyses of the first look. For dynamic (A) and static (B) condition of the face scanning task, proportions of first look on the eyes

(FLoE) were calculated by dividing the number of trials participants fixated on the eyes before the mouth by the total number of valid trials. As in the main analyses, each of 8 trials were excluded if less than one second of data was accumulated. In the dynamic condition, FLoE was significantly smaller in the SIBP group than in control group ($F(1, 35) = 4.18$, $p = .049$, $\eta_p^2 = .11$), suggesting that SIBP group were less likely to fixate on the eyes before the mouth compared to the control groups when they observe dynamic face. FLoE did not differ between groups in the static condition ($F(1, 35) = .44$, $p = .510$, $\eta_p^2 = .01$). Neither the main effect of visit nor the interaction between the group and the visit reached significance in either dynamic or static condition (all $F < 1.70$, all $p > .180$, all $\eta_p^2 < .06$).
C-F: Results of subgroup analyses of eye-tracking measurements in sighted infants of blind parents (SIBP), which contrast those children with blind fathers (n = 6) and those with sighted fathers (n = 8) in (C,D) the face scanning task, eye–mouth index (EMI) in (C) dynamic and in (D) static conditions, in (E, F) the gaze-following task, (E) differential looking score (DLS) and (F) looking time. Note that we did not find any significant main effect of group (all $F < 2.24$, all $p > .166$, all $\eta_p^2 < .18$) or group by visit interaction (all $F < 3.71$, all $p > .083$, all $\eta_p^2 < .27$) on the variables which showed significant difference between SIBP and controls. The main effect of group ($F(1, 8) = 19.03$, $p = .002$, $\eta_p^2 = .70$) and the interaction between group and visit ($F(1, 8) = 5.38$, $p = .049$, $\eta_p^2 = .40$) were significant in DLS, suggesting that SIBP with both blind mother and father show more frequent gaze following at time 1, but no further interpretations were made as we did not find any group differences between SIBP and controls on this variable.
The upper whisker extends from the hinge to the highest value that is within 1.5 * IQR of the hinge (where IQR is the inter-quartile range, or distance between the first and third quartiles). The lower whisker extends from the hinge to the lowest value within 1.5 * IQR of the hinge. Individual data points were also plotted on top of the box plots.
